# Histologic and Genotypic Characterization of Lung Cancer in the Inuit Population of the Eastern Canadian Arctic

**DOI:** 10.3390/curroncol29050258

**Published:** 2022-04-29

**Authors:** Glenwood D. Goss, Johanna N. Spaans, David Huntsman, Timothy Asmis, Natalie M. Andrews Wright, Marc Duciaume, Pardeep Kaurah, Ruth R. Miller, Shantanu Banerji, Harmanjatinder S. Sekhon, Marcio M. Gomes

**Affiliations:** 1 The Ottawa Hospital Research Institute, 501 Smyth Rd, Ottawa, ON K1H 8L6, Canada; johanna.spaans1@gmail.com (J.N.S.); nawright@ohri.ca (N.M.A.W.); marcduciaume1@gmail.com (M.D.); hsekhon@eorla.ca (H.S.S.); 2 Division of Medical Oncology, Department of Medicine, The Ottawa Hospital Cancer Center and the University of Ottawa, ON K1N 6N5, Canada; tasmis@toh.ca; 3 Imagia Canexia Health, Suite #204 Donald Rix Building, 2389 Health Sciences Mall, Vancouver, BC V6T 1Z3, Canada; dhuntsman@canexiahealth.com (D.H.); pkaurah@bccancer.bc.ca (P.K.); rmiller@canexiahealth.com (R.R.M.); 4 Department of Pathology and Laboratory Medicine, University of British Columbia, Vancouver, BC V6T 1Z4, Canada; 5 Department of Medical Genetics, Faculty of Medicine, University of British Columbia, and Hereditary Cancer Program, BC Cancer, Vancouver, BC V5Z 4E6, Canada; 6 CancerCare Manitoba Research Institute, Rady Faculty of Health Sciences, University of Manitoba, Winnipeg, MB R3E 0V9, Canada; sbanerji@cancercare.mb.ca; 7 Department of Pathology, The Ottawa Hospital and the University of Ottawa, Ottawa, ON K1N 6N5, Canada

**Keywords:** lung cancer, Inuit, histologic, genomic

## Abstract

Inuit are the Indigenous Arctic peoples and residents of the Canadian territory of Nunavut who have the highest global rate of lung cancer. Given lung cancer’s mortality, histological and genomic characterization was undertaken to better understand the disease biology. We retrospectively studied all Inuit cases from Nunavut’s Qikiqtani (Baffin) region, referred to the Ottawa Hospital Cancer Center between 2001 and 2011. Demographics were compiled from medical records and tumor samples underwent pathologic/histologic confirmation. Tumors were analyzed by next generation sequencing (NGS) with a cancer hotspot mutation panel. Of 98 patients, the median age was 66 years and 61% were male. Tobacco use was reported in 87%, and 69% had a history of lung disease (tuberculosis or other). Histological types were: non-small cell lung carcinoma (NSCLC), 81%; small cell lung carcinoma, 16%. Squamous cell carcinoma (SCC) represented 65% of NSCLC. NGS on 55 samples demonstrated mutation rates similar to public lung cancer datasets. In SCC, the *STK11* F354L mutation was observed at higher frequency than previously reported. This is the first study to characterize the histologic/genomic profiles of lung cancer in this population. A high incidence of SCC, and an elevated rate of *STK11* mutations distinguishes this group from the North American population.

## 1. Introduction

Inuit are the Indigenous peoples of the Arctic who, in Canada, primarily inhabit the arctic and sub-arctic regions of Nunavut, Nunavik (northern Quebec), Nunatsiavut (northern Labrador) and the Inuvialuit Settlement Region (northern Northwest Territories), known as Inuit Nunangat [1]. With a lung cancer age-standardized incidence ratio of 145.8/100,000 in men and 155.3/100,000 in women, residents of Nunavut have lung cancer rates 2.98 times and 4.52 times (in men and women, respectively) the national average [2]. In fact, Nunavut has the highest incidence rate of lung cancer in the world which presents an important public health concern [2,3].

The Ottawa Hospital Cancer Center (TOHCC) services a population of 1.6 million people [4], including approximately 19,000 people (2016 data) [5] who live in the Qikiqtani (Baffin) Region of Eastern Nunavut (personal communication, Cancer Care Ontario). Since 1998, TOHCC has been the referral cancer center for the Baffin Region. The elevated rate of lung cancer among Inuit is reflected in the number of patients referred to TOHCC. From 2000 to 2010 lung cancer represented 48.6% of all Inuit cancer referrals from Nunavut [6], whereas in the total population serviced by TOHCC, lung cancer cases only represented 13% of referrals (unpublished Health Records data).

Lung cancer can be divided into two primary histological subgroups: small cell lung carcinoma (SCLC) and non-small cell lung carcinoma (NSCLC), which represent 15% and 85% of annual lung cancer diagnoses, respectively [7]. The main histological subtypes of NSCLC are adenocarcinoma and squamous cell carcinoma (SCC) [8]. In the general North American population, adenocarcinoma is the most common histological sub-type, representing 44% of newly diagnosed lung cancers while SCCs account for 26% of diagnoses and are most frequently associated with smokers and the male sex [7,9], though the proportional representation of each histology is evolving as smoking rates decline and diagnostic criteria are updated [10,11]. These histological subtypes are further molecularly subclassified by the presence of dominant oncogenic driver mutations [12,13]. Adenocarcinoma in particular has the most identified actionable mutations, as evidenced by the approval of multiple targeted therapies directed against *EGFR*, *ALK*, *ROS1*, *BRAF* V600 and *NTRK* aberrations [14,15,16,17,18]. SCC harbors distinct genetic alterations compared to adenocarcinoma with fewer known oncogenic driver mutations [12].

In lung cancer, ethnicity-based prevalence differences of some oncogenic mutations have been documented, most notably *EGFR* mutation frequency in adenocarcinoma of the lung [19,20]. Specifically, while *EGFR* mutations occur in approximately 15% of adenocarcinoma NSCLC in Caucasian populations in Canada, USA and Europe, this mutation is far more frequent in the East Asian population, with a prevalence of 40–50% (reviewed in [19]). Molecular characterization of lung cancer in ethnic minorities is therefore important [21] and to our knowledge, no study has characterized lung cancer in the Inuit population. We hypothesized that ethnic-specific molecular differences exist within lung cancers from the Inuit population and interrogated their histological and genomic features. Study objectives include: describing the histology of lung cancer among Canadian Inuit (frequency), establishing prevalence of common mutations using next generation sequencing (NGS) and correlating cancer type, histology, molecular profile with other clinical and demographic characteristics.

## 2. Materials and Methods

### 2.1. Patients and Study Design

This is a retrospective 10-year review of the demographic, histologic and molecular characteristics of lung cancer among Canadian Inuit diagnosed and referred for cancer treatment to TOHCC. Given the limited number of Inuit lung cancer patients, all available cases meeting inclusion criteria were included and cases have some overlap with a previous report [6]. Patients from The Ottawa Hospital Cancer Center were included if they were of Inuit ethnicity, had been diagnosed with lung cancer (any histology) between 2001 to 2011 and were deceased. To ensure Inuit-specific data, only lung cancer cases among confirmed Canadian Inuit land claims beneficiaries were included in the review, which was established based on Nunavut health insurance card coding information. Socio-demographic (age, sex, smoking history), diagnostic (type and stage of cancer), treatment-related information (prior surgery, chemotherapy, radiotherapy) and other clinically relevant information including co-morbidities (chronic obstructive pulmonary disease (COPD), tuberculosis), history of malignancy and occupational exposure data were collected for all identified patients from their medical charts. This study was conducted in accordance with the Canadian Tri-Council policy statement for the ethical conduct of research involving Aboriginal Peoples and in accordance with the Declaration of Helsinki [22,23]. The study was developed in consultation with the Government of Nunavut and Nunavut Tunngavik Inc. The protocol, including specimen collection and waiver of consent for deceased patients, was approved by The Ottawa Health Science Network Research Ethics Board (#20130646-01H) and registered with the Nunavut Research Institute.

### 2.2. Histology

All tumors were reclassified according to the 4th edition of the WHO classification of lung tumors [8]. Pathology review of all available archival tissue samples was performed by two pulmonary pathologists (M.G. and H.S.). Immunohistochemistry and mucin stain were performed in cases with adequate archival formalin-fixed paraffin-embedded (FFPE) tissue blocks on a case-by-case approach following recommended diagnostic algorithm: (1) for specific histological subtyping in NSCLC cases lacking definite morphological differentiation; and (2) for confirming or ruling out neuroendocrine differentiation when morphologically suspected [24]. In patients lacking tumor sample for pathology review, histological features described on the original pathology report were used for reclassification, whenever feasible.

### 2.3. Molecular Analysis

Molecular analysis was undertaken on all tumors for which there was adequate DNA material from either formalin fixed paraffin-embedded (FFPE) archival tumor blocks or cell blocks from cytology specimens. The analyses were performed by Contextual Genomics Inc.^®^, under their National Access Program [25]. The FIND IT^TM^ (CG001) version 2.7 hotspot mutation panel developed by Imagia Canexia Health (previously Contextual Genomics) was used and the specific regions targeted for sequencing are provided as Supplemental Material (Table S1). Following amplification, enriched DNA libraries were sequenced on the Illumina MiSeq [26]. Sequence data analysis was performed using a validated, custom-built bioinformatics pipeline MutationSeq [27], proven to be suited for targeted single nucleotide variation (SNV) and indel detection. Sequence data (fastq files) generated by the Illumina MiSeq were mapped to the reference genome (hg19) and SAMtools and bamUtils were used to remove poor quality reads. MutationSeq and Strelka algorithms were then used to detect SNVs and indels, respectively. Finally, the functional effect of the detected high confidence mutations was annotated using SnpEff. The data were subject to stringent quality control at each analysis step. Results were not reported for samples that failed quality control (QC) with <500× coverage in ≥5 amplicons or <1000× coverage in ≥10 amplicons. Only samples with mutations in regions of interest with a minimum of 500× coverage, high probability (≥0.9) and limits of detection (allele ratio ≥ 5%) are reported. The Quantitative Multiplex DNA Reference Standard (Horizon Diagnostics) and AcroMetrix Oncology Hotspot Control (Life Technologies) were used as positive controls and the NA01953 cell line (Coriell Biorepositories) served as a negative control and a normal reference.

### 2.4. CBioPortal

For each histological subtype the following studies were queried from cBioportal [28]: Lung Adenocarcinoma: TSP; Nature 2008, TCGA; Nature 2014; MSKCC 2015; and Broad, Cell 2012 (*N* = 610). Lung Squamous Cell Carcinoma: TCGA, Nature 2012 (*N* = 178). Small Cell Lung Cancer: CLCGP, Nat Genet 2012; Johns Hopkins, Nat Genet 2012; and U Cologne, Nature 2015 (*N* = 181). All mutations in genes covered by the FIND IT^TM^ v2.7 panel were included (*AKT1*, *ALK*, *AR*, *BRAF*, *CDKN2A*, *CTNNB1*, *EGFR*, *ERBB2*, *ESR1*, *FGFR1*, *FGFR2*, *GNA11*, *GNAQ*, *GNAS*, *HRAS*, *IDH1*, *IDH2*, *JAK1*, *KIT*, *KRAS*, *MAP2K1*, *MAP2K2*, *MET*, *NRAS*, *PDGFRA*, *PIK3CA*, *PTEN*, *RET*, *STK11*).

### 2.5. Statistical Considerations

**Figure 2:**
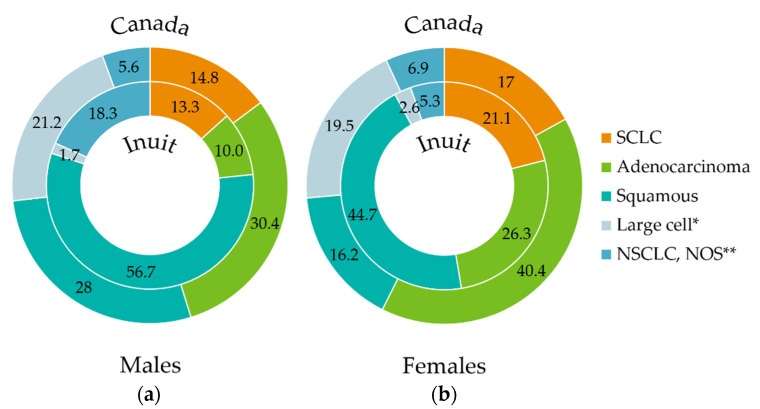
Percentage distribution of lung cancer histologies (inner circle) in Inuit males (**a**) and females (**b**) within the study cohort (2001–2011) compared to reported histological proportions in Canada (1998–2002) (outer circle, adapted from [29]). Data labels represent percentages (%). Archived tumor tissue from study participants was re-assessed by the study pathologist (M.G.) to confirm histological diagnosis, using the 4th Edition of the WHO classification of tumors of the lung [8]. In patients with no tissue available for review, the original pathological diagnosis was kept, the large proportion of NSCLC, NOS is attributable to small biopsies with insufficient tissue for complete pathological testing (i.e., stains). For the Canadian data, the individual age-standardized incidence ratios of each histology were calculated as a proportion of the total age-standardized incidence ratio of all histologies and normalized to a percentage value. * “Large cell” designation represents large cell neuroendocrine carcinoma (LCNEC) in the Inuit cohort, whereas in the Canadian cohort, it represents large cell carcinoma as classified at the time of data collection, prior to the 4^th^ Edition of WHO classification of tumors of the lung [8]. ** Other/NOS includes NSCLC NOS and carcinoma NOS. SCLC, Small cell lung cancer; NSCLC, Non-small cell lung cancer; NOS, not otherwise specified.

Descriptive statistics were used to define age, tobacco use, history of lung disease (including tuberculosis, pneumonia, COPD, etc.), stage of disease at diagnosis, and histological type and subtype by gender. The clinical and molecular data were correlated to identify common oncogenic mutations by histology subtype. When citing Canadian histological distribution in Figure 2, age-standardized incidence rates from the International Agency for Research on Cancer [29] were expressed as a percentage based on their proportion of the total incidence, for each histology.

For genomic analysis, percent of mutations was calculated using all mutations including mutations in samples with more than one mutation (in the numerator and denominator), for mutations identified by FIND IT^TM^, this included SNVs and indels. Associations of genomic alteration frequency in specific genes between the study population and cBioPortal were conducted using a Fisher’s exact test. Given the small sample size, *p* values should be interpreted with caution.

## 3. Results

### 3.1. Participant Selection and Study Flow

Between 2001 and 2011, 109 patients of Inuit ethnicity were seen at the Ottawa Hospital Cancer Center with a diagnosis of lung cancer. As per approval from the Research Ethics Board (REB), only deceased patients were considered for histological and mutational analysis (*n* = 98). Histological subtype is reported on all 98 samples, although only 89 samples could be reviewed as 9 cases did not have archival material available. For these cases, the original pathological diagnosis is reported.

**Figure 1:**
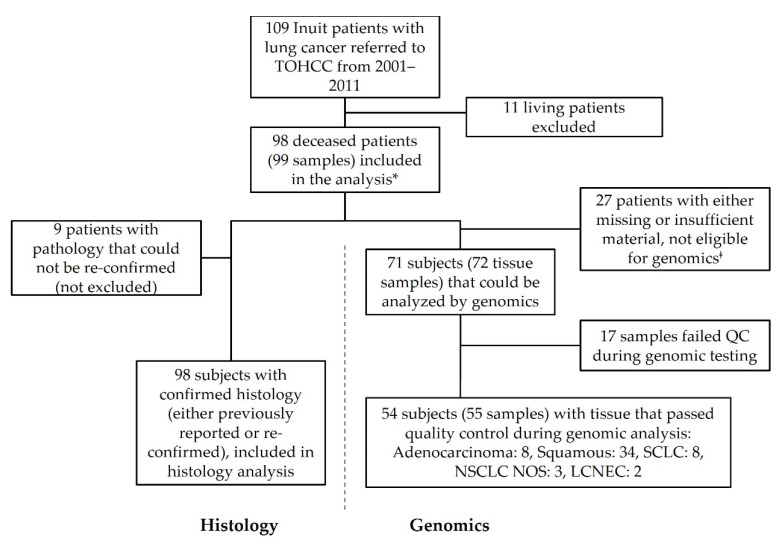
Flow diagram of study subjects. Inuit patients with lung cancer referred to the Ottawa Hospital Cancer Center from the Baffin region of Nunavut between 2001 and 2011. Subjects were assessed for inclusion/exclusion, with the primary criteria being survival status. Pathology specimens sought from all cases were reviewed when available and for those patients with sufficient tissue, molecular analysis was conducted. * One case had two primary lung cancers that were considered as distinct tumors though they originated from the same patient. ^‡^ These cases included blocks not available, blocks with insufficient tissue and blocks that failed H&E review at Contextual Genomics (now Imagia Canexia Health). LCNEC, large cell neuroendocrine carcinoma; NOS, not otherwise specified; NSCLC, non-small cell lung cancer; QC, quality control; SCLC, small cell lung cancer; TOHCC, The Ottawa Hospital Cancer Center.

Of the total study population, 26 patients had insufficient archival tissue for molecular analysis and 2 samples failed pathology review (H&E) at Contextual Genomics (now Imagia Canexia Health), one of which was a duplicate sample and therefore is dropped from the calculations. Therefore, 72 samples were interrogated by NGS for hotspot mutations. Of these, 55 passed quality control (QC) and reported herein. A total of 17 cases failed QC and were excluded. It should be noted that the discrepancy in the number of subjects (*n* = 54) and the number of tested samples (*n* = 55) is due to one subject with two primary tumors that were both included (Figure 1).

### 3.2. Patient Demographics

Baseline population characteristics of the 98 patients are shown in Table 1. The median age was 66 years and 61% were male. A large majority (85/98, 87%) were current or ever smokers and only one subject (1%) was a never-smoker. Smoking status was unknown in 12% of cases. History of lung disease was documented in 68/98 (69.4%) cases (40/60 (67%) men, 28/38 (74%) women), 1 case (male) was not reported. Tuberculosis was the most common lung disease, documented in 46/98 (47%) of patients overall (27/60, 45%, in males and 19/38, 50% in females). A history of other lung disease (including: pneumonia, COPD, asthma, fibrosis, bronchiectasis, emphysema or interstitial lung disease) was documented in 51/98 (52%) patients. Excluding SCLC cases, diagnosis of incurable disease was common, with 26/52 (50%) men and 12/30 (40%) of women having stage IV disease at the time of diagnosis.

### 3.3. SCC of the Lung Is the Most Common Histological Subtype in This Cohort

In line with the primary outcome, we conducted pathology review of lung tumors specimens to examine differences in the frequency of histological subtypes. These data are presented in comparison to previously reported histological distributions among Canadian cases of lung cancer (Figure 2) [30]. SCLC was represented in 16% of cases (13% of men and 21% of women) and NSCLC was represented in 81% (83% of men and 76% of women). Large-cell neuroendocrine carcinoma (LCNEC) was found in two cases (2%) (one male, one female).

Histology could not be specified (carcinoma not otherwise specified (NOS)) for one case with limited tissue sample. Within NSCLC, SCC was the most common histological subtype, representing 65% of all cases. This diagnosis was most common in men where it represented 68% of NSCLC and 57% of all lung cancers. In women, SCC accounted for 59% of NSCLC and 45% of all lung cancers. Within NSCLC, adenocarcinoma represented a larger proportion of cases in women (34% of NSCLC and 26% of all lung cancer cases) than in men (12% of NSCLC and 10% of all lung cancer cases). There were 12 cases in which the diagnosis of NSCLC could not be further specified.

### 3.4. Genomic Analysis Highlights Missense Mutations in STK11, PIK3CA and KRAS

**Figure 3:**
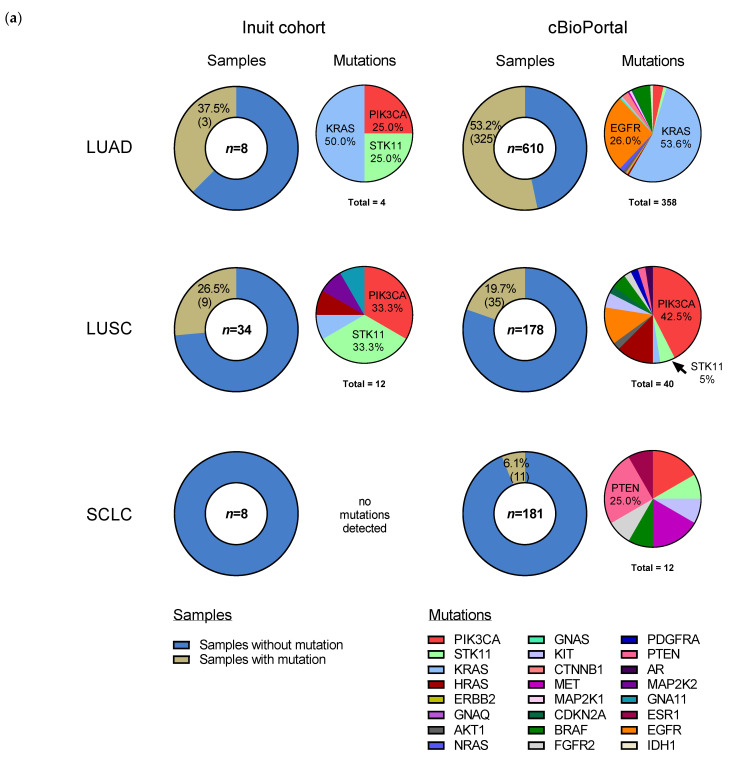

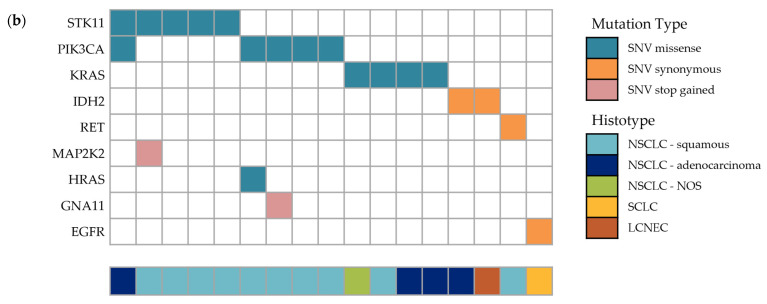
Somatic mutations in the specimens of study cohort of Inuit lung cancer patients (2001–2011). (**a**) Comparison of the prevalence of non-synonymous mutations in lung cancer between the Inuit and cBioPortal cohorts, by sample (left chart of respective column) and gene (right chart of respective column), in the most common histological subtypes. Mutation frequency by sample in study specimens (*n* = 50, 5 not shown: 3 NSCLC NOS and 2 LCNEC) is compared to cBioPortal [30] dataset (*n* = 969; LUAD = 610, LUSCs = 178, SCLC = 181), where “*n*” denotes the number of samples evaluated in each histological subtype. Mutation frequency of individual genes are represented to the right of the sample charts. “Total” indicates the total number of mutations in samples with ≥ 1 mutation. Co-occurring mutations account for the discrepancy between the number of total mutations and the number of samples with mutations. See Table S2 for the complete list of mutations detected in the Inuit cohort. Within mutations from cBio portal, 18% of genes mutated in LUAD, 25% of genes mutated in LUSC and 17% of genes mutated in SCLC were co-occurring. Enriched DNA libraries were sequenced using the Illumina MiSeq. Sequence data analysis was performed using a validated, custom built bioinformatics pipeline, using MutationSeq [27]. (**b**) Oncoplot summary of detected mutations in all samples containing SNVs. SNV: single nucleotide variant, LCNEC: large-cell neuroendocrine carcinoma, LUAD: lung adenocarcinoma, LUSC: lung squamous cell carcinoma, NSCLC: non-small cell lung cancer, SCLC: small cell lung cancer.

As planned, we used NGS to characterize the prevalence of common SNVs and short insertion/deletions in Inuit lung cancer specimens (Figure 3). Analysis of the FIND ITTM hotspot mutation panel (Table S1) was successfully conducted on 55 samples from 54 cases (as 1 case had 2 primary lung cancers separated by 4 years (2003 and 2007) that were evaluated as distinct specimens). Of note, this panel does not detect mutations in *TP53*, the most frequently mutated gene across lung cancer subtypes.

Fifty cases are illustrated in Figure 3a (low frequency histologies, carcinoma NOS (*n* = 3) and LCNEC (*n* = 2), are excluded). Overall, there were 17 genetic alterations (insertions/deletions and SNVs), excluding synonymous mutations, found in 12 of 55 samples (21.8%), which include: STK11 (in 5 samples; 9.1%), PIK3CA (in 5 samples; 9.1%), KRAS (in 4 samples; 7.3%), and GNA11, HRAS and MAP2K2 in 1 sample each. Four cases had co-occurring mutations: GNA11-PIK3CA, HRAS-PIK3CA, STK11-PIK3CA and STK11-MAP2K2. Synonymous variants were found in IDH2 (two cases), RET and EGFR, and are shown in Figure 3b. Non-synonymous (missense) SNVs accounted for nearly all detected alterations, however *MAP2K2* and *GNA11* harbored a non-sense (stop) mutation. Recurring SNV included *STK11* F354L (five samples), *PIK3CA* E545K (five samples), *HRAS/KRAS* G12S/V (three samples) and *KRAS* Q61H (two samples). The full list of detected genetic alterations is found in Table S2.

### 3.5. Somatic Mutation Profile from Genomic Analysis Varies by Histology and Is Similar to cBioPortal Data

For each histological subtype, we examined overall mutation rates of tumors from our study population and compared them to cBioPortal, a large electronic repository of cancer genomics data sets [28,30] (Figure 3a). LCNEC (*n* = 2) and NSCLC NOS (*n* = 3) were excluded from the graphical representation as their small sample size limits meaningful interpretation. In the Inuit cohort, excluding synonymous mutations, we detected somatic alterations in 37.5% (3/8) of adenocarcinoma samples, 26.5% (9/34) of SCC samples and 0% (0/8) of SCLC samples, compared to cBioPortal where mutation rates for these histological subtypes were, respectively, 53.2%, 19.7% and 6.1%. The difference in overall somatic mutation rate for each histological subtype did not differ statistically between the Inuit and cBioPortal cohorts (Fisher’s exact test), though the small sample size from our study likely precludes meaningful analysis. Three genes accounted for 14 of 17 (82%) detected missense mutations: *STK11*, *PIK3CA* and *KRAS* (Figure 3a). The three (3) adenocarcinoma cases with detected mutations included: *KRAS* (2) and *PIK3CA-STK11* (1). Each mutated gene is expressed as a percentage of all mutations found (Figure 3a), and thus does not exactly match the number of samples, as some samples had co-occurring mutations. *KRAS* mutations represented 50% (2/4) of all mutations in the Inuit adenocarcinoma samples, compared to 53.6% (192/358) of cBioPortal adenocarcinoma mutations. Non-synonymous *EGFR* mutations were not detected in any Inuit adenocarcinoma case, whereas they represented 26.0% of all mutations in the cBioPortal cohort. In the nine (9) SCC cases with detected mutations, the most common, *STK11* F354L and *PIK3CA* E545K, were identified in four samples each, with *KRAS*, *HRAS*, *MAP2K2* and *GNA11* (one each) making up the remaining detected mutations. In three (3) of those individuals, the mutations were co-occurring (*STK11*-*MAP2K2* (stop), *PIK3CA-GNA11* (stop) and *PIK3CA-HRAS*). *PIK3CA* mutations represented 33.3% of mutations in the Inuit cohort (4/12) and 42.5% (17/40) of mutations in cBioPortal (*p* = 0.7407, Fisher’s exact test). Interestingly, while mutations in *STK11* represented 33.3% (4/12) of mutations in the Inuit cohort, they accounted for only 5% (2/40) of cBioPortal samples (neither of which were the F354L variant), (*p* = 0.0206, Fisher’s exact test). One of three NSCLC NOS cases had a non-synonymous *KRAS* mutation. We detected no non-synonymous hotspot mutations in any SCLC case tested (0/8), although the FIND IT^TM^ panel did not include *TP53* and *RB1*, the genes most commonly mutated in this subtype. There were no non-synonymous mutations detected in cases of LCNEC (0/2). Across all Inuit samples, *STK11* and *PIK3CA* were found to be mutated exclusively in the F354L (1062C > G) and E545K (1633G > A) positions, respectively.

Thus, differences in overall mutation rates in each histological subtype did not reach statistical significance when compared to the overall mutation rate per histology in cBio-Portal. However, an apparent increase in the frequency of the F354L *STK11* mutation was noted though we encourage cautious interpretation of this finding due to small sample sizes.

## 4. Discussion

It is known that the Inuit population of Nunavut is unique in that it has among the highest incidence rates of lung cancer in the world, an elevated rate of tobacco usage and a high incidence of lung disease, including high rates of tuberculosis. In this study, we document the demographic and genomic characteristics of a cohort of Inuit lung cancer patients from the Baffin Region of Nunavut, Canada, referred to TOHCC between 2001 and 2011. We reviewed the pathologic features and tumor genetics using a NGS panel of selected common cancer hotspots and insertions/deletions. Our important histological finding is that, distinct from the current North American population, there is a higher proportion of SCC tumors than other histological subtypes among Inuit. Our genomic analysis demonstrates a relatively similar pattern of total alterations compared to large global data sets. However there appears to be an increased genomic prevalence of F354L *STK11* mutations in cases of SCC, a finding unique to this population.

Demographically, the median age of the study cohort at the time of lung cancer diagnosis was 4 years younger than the North American average of 70. Distribution by sex was uneven, men represented 61.2% of cases in our cohort, which is similar to the 2002 Canadian data, where lung cancer cases were 60.8% male [31].

### 4.1. The Elevated Prevalence of SCC Histology Is Consistent with Determinants of Health Affecting This Cohort/Population

To our knowledge, this is the first study to histologically characterize lung cancer among a cohort of Canadian Inuit, who have some of the highest lung cancer rates worldwide [2]. While the relative proportions of SCLC and NSCLC noted in our study was comparable to elsewhere in North America, the histological distribution within NSCLC differed significantly from previous North American reports [32]. In our studied population, adenocarcinoma is not the dominant subtype and there were more than three times as many squamous cell carcinomas (51 cases) as adenocarcinomas (16 cases). This important representation of SCC in the population, rather than adenocarcinoma, indicates that currently actionable mutations common in adenocarcinoma are less likely to be found in this group.

The rate of SCC in this cohort is considerably higher than previously reported Canadian rates (57% vs. 31.7% in men and 45% vs. 21.1% in women, respectively) as a percentage of all lung cancers [32]. The Territory of Nunavut, however, has reported that SCC was the most commonly diagnosed lung cancer histology from 2000 to 2010 [33]. In North American males, adenocarcinoma is the most commonly diagnosed histology, whereas SCC is the most common in males from Korea, France and the United Kingdom (UK) [31]. In a retrospective study of SEER data, women were also more frequently diagnosed with adenocarcinoma than SCC, and this is also shown to be true on a global level [7,31]. The UK is an exception, where 28% (which represented the highest proportion of cases) of females were diagnosed with SCC [31]. This is in contrast to our study where 45% of Inuit females were diagnosed with SCC and 26% were diagnosed with adenocarcinoma, showing that in our cohort, women have a much higher rate of SCC and lower rate of adenocarcinoma than elsewhere in the world.

The relative proportion of SCCs to other histological subtypes reported herein is similar to what has been reported for smokers [34]. As smoking is the most important risk factor for the development of lung cancer, especially for SCC and SCLC subtypes, elevated smoking rates among Inuit are thought to be contributing to the growing incidence of lung cancer in this population [35,36]. As of 2012, 52% of Inuit aged 15 and older smoked cigarettes daily, over three times the national (Canadian) rate (16%), and this rate has since increased [37,38]. This is in contrast with the rest of Canada, where both smoking and SCC rates are declining [32,39]. As there is a roughly 30 year latency period between peak tobacco exposure and lung cancer incidence, lung cancer will remain a critical health concern for Nunavut for years to come [40].

Other lung diseases are also likely contributing factors to our findings. Tuberculosis (TB) remains an important health concern in Nunavut as recently published figures place the age-standardized rate of TB in Nunavut at almost 40 times the Canadian average [41]. In addition to TB, other respiratory conditions, such as COPD, bronchitis and pneumonia, were common in the study population. There is a positive association between lung diseases (including TB, COPD, chronic bronchitis and pneumonia) and lung cancer risk [42,43]. A prospective case-matched study of 113 patients with COPD over 10 years, controlled for age, sex, occupation and smoking history, showed that the probability of developing lung cancer was significantly higher in patients with COPD [44]. Similarly, a longitudinal cohort study of over 700,000 Taiwanese showed an incidence of lung cancer approximately 11-fold higher in patients with tuberculosis, when controlled for demographic factors and comorbidities [45]. Regarding the impact of prior lung disease on lung cancer histology, there is some evidence that TB infection can predispose lung tissues to SCC, as demonstrated in a mouse model study by Nalbandian and colleagues [46].

Taken together, our data have shown that SCC is the dominant histological subtype in the Inuit of the Baffin region of Nunavut, Canada, and that this finding may be influenced by important factors in this population such as smoking, high rates of TB and other lung diseases.

### 4.2. Genomic Analysis Shows Similar Mutational Profiles in Each Histological Subtype When Compared to cBioPortal Data

Genomic analysis of the Inuit lung cancer samples revealed a mutational landscape that was relatively similar to those found in large datasets (cBioPortal). While overall, the total mutational burden in each of the histological subtypes evaluated (adenocarcinoma, SCC and SCLC) was not significantly different from the cBioPortal population, there were qualitative differences within histological subtypes. Our small sample size limits the direct comparison between the mutation rates of specific genes in the Inuit population compared to the cBioPortal data but this hypothesis-generating approach identified genes of interest.

In the adenocarcinoma group, it is interesting that we did not find any non-synonymous *EGFR* mutations, typically found in 15% of the (global) sample repertoire of cBioPortal. This may be due to our small sample size not being truly representative. The prevalence of other top-mutated genes, *PIK3CA* in SCC and *KRAS* in adenocarcinoma, from Inuit cases is comparable to global cases seen in cBioPortal [12] and raises the possibility that novel inhibitors of phosphatidylinositol 3-kinase (PI3K) and KRAS may have value in this population if ongoing clinical trials report positive results in the future.

Lastly, our finding of no non-synonymous mutations in SCLC is in keeping with the lack of known driver mutations in this histology.

### 4.3. STK11 F354L Mutation

*STK11* mutations are commonly found in lung adenocarcinoma, but not in other sporadic tumors [47,48]. In patient samples, *STK11* function is often altered either by inactivating point mutations, reduced gene expression or chromosomal loss in 4–34% of tested human adenocarcinomas of the lung, with the percentage of somatic mutations being described as lowest in Asian cohorts and highest in Caucasians [13,49,50,51,52]. *STK11* mutations, however, have not frequently been reported in SCC or other lung tumor subtypes [12,53]. It was therefore interesting to find a relatively larger number of mutations in our SCC cohort than previously reported.

The F354L polymorphism has previously been described in lung and other malignancies and results suggest that the F354L allele is a germline variant rather than an oncogenic mutation [54]. Germline DNA testing was not conducted as part of this study. One study has reported *STK11* F354L in lung cancers, but not in healthy tissue [55]. Gene mapping indicates a c-terminus location for the amino acid at residue 354, outside the STK11 catalytic domain, and several studies have shown that the kinase activity of F354L allele, and its ability to form a complex with STRAD and MO25, are preserved compared to the wild-type protein [54,56,57]. However, the c-terminus can be an important area for post-translational modifications and there are conflicting data on the impact of this mutation on AMPK-phosphorylation, with one group reporting a decrease in AMPK phosphorylation, and consequently on downstream pathway activation, and another showing no effect on AMPK phosphorylation either in the presence or absence of WT protein [56,57]. While it is generally accepted that the *STK11* F354L mutation is a germline variant, more comprehensive genomic studies could shed light on remaining uncertainty.

It is important to recognize the areas of potential bias in this retrospective study apart from sample size. Areas to be highlighted include patients analyzed as part of this study were both referred and travelled to Ottawa, thus excluding those who were not diagnosed, who were not referred, who did not travel to Ottawa, or who were of Inuit ethnicity but residing outside the Baffin region. A recent report indicates up to 30% of cancer patients from Nunavut are not referred to Ottawa, though this research did not examine the specific causes [6]. Therefore, differences in demographic characteristics must be evaluated with this in mind.

## 5. Conclusions

This study has shown that the histological characteristics of lung cancer among Canadian Inuit are different than the general Canadian and North American populations, with SCC being far more frequent in this study cohort. This initial NGS analysis, although limited in scope, suggests that the genetic profile of oncogenic drivers of lung cancers in Inuit is similar to the rest of the North American population. However, we identified a higher rate of *STK11* mutations in squamous cell carcinoma in the Inuit tumors than previously reported and this will need to be confirmed with further studies. The most commonly identified mutations in oncogenic drivers—in *STK11*, *PIK3CA* and *KRAS*—are candidates for targeted therapy. In conclusion, demographic findings in this study cohort highlight the necessity for the ongoing smoking cessation programs in the Qikiqtani (Baffin) Region of Eastern Nunavut.

## Figures and Tables

**Table 1:** Demographic and pathological baseline characteristics in Inuit from the Eastern Canadian Arctic referred to The Ottawa Hospital Cancer Centre between 2001 and 2011 included in the study cohort.

Baseline Characteristics	Men *n* = 60		Women *n* = 38		Total *n* = 98	
Age, y						
Median	66		66		66	
Range	35–82		44–87		35–87	
Tobacco use history, *n* (%)						
Current	35	(58)	18	(47)	53	(54)
Previous	18	(30)	14	(37)	32	(33)
Never	0	(0)	1	(3)	1	(1)
Unknown	7	(12)	5	(13)	12	(12)
History of lung disease, *n* (%)						
Reported (any)	40	(67)	28	(74)	68	(69)
Tuberculosis	27	(45)	19	(50)	46	(47)
Other lung disease (i.e., pneumonia, COPD, etc.)	27	(45)	24	(63)	51	(52)
Stage at diagnosis, *n* (%)						
Solid tumor ^1^, *n*	52		30		82	
I	3	(6)	6	(20)	9	(11)
II	2	(4)	1	(3)	3	(4)
III	20	(38)	10	(33)	30	(37)
IV	26	(50)	12	(40)	38	(46)
Unknown	1	(2)	1	(3)	2	(2)
Pathology/histology, *n* (%)						
Non-Small Cell Lung Cancer (NSCLC)	50	(83)	29	(76)	79	(81)
Adenocarcinoma	6	(10)	10	(26)	16	(16)
Squamous Cell	34	(57)	17	(45)	51	(52)
NOS	10	(17)	2	(5)	12	(12)
Small Cell NEC (SCLC)	8	(13)	8	(21)	16	(16)
Large Cell NEC (LCNEC)	1	(2)	1	(3)	2	(2)
Carcinoma, undifferentiated	1	(2)	0	(0)	1	(1)

^1^ Stage at diagnosis excludes SCLC cases, number of cases included is therefore indicated. COPD, chronic obstructive pulmonary disease; NOS, not otherwise specified; NEC, neuroendocrine carcinoma. Due to rounding, not all sums of percentages equate exactly 100%.

## Data Availability

The data presented in this study are available on request from the corresponding author. The data are not publicly available in accordance with Research Ethics Board approval.

## Supplementary Materials

The following supporting information can be downloaded at: https://www.mdpi.com/article/10.3390/curroncol29050258/s1, Table S1: FIND ITTM Hotspot mutation panel and queried CBioPortal gene list; Table S2: Summary of detected genetic alterations.
