# Supplementary material for: Histologic and Genotypic Characterization of Lung Cancer in the Inuit Population of the Eastern Canadian Arctic
Source: Curr Oncol. 2022 Apr 29;29(5):3171–86. doi: 10.3390/curroncol29050258 (PMC9139845; doi:10.3390/curroncol29050258)
Supplement: Supplementary file 1 [file curroncol-29-00258-s001.zip › curroncol-1685434-supplementary.pdf]

Supplementary Material, Table S1: FIND ITTM Hotspot mutation panel and queried CBioPortal gene list

(a)

Gene	Codon / Exon
**AKT1**	E17
**ALK**	T1151, L1152, C1156, F1174, L1196, G1269, R1275
**AR**	S741, W742, H875, Q876, T878
**BRAF**	Q201, G466, G469, Y472, D594, G596, L597, V600
**CDKN2A**	R58
**CTNNB1**	S37, T41, S45
**EGFR**	Exons 18, 19, 20, 21
**ERBB2**	G309, S310
**ESR1**	V534, P535, L536, Y537, D538
**FGFR1**	N546, K656
**FGFR2**	S252, P253, N549, K659
**GNA11**	Q209
**GNAQ**	Q209
**GNAS**	R201
**HRAS**	G12, G13, Q61

Gene	Codon / Exon
**IDH1**	R132
**IDH2**	R140, R172
**JAK1**	V658, S703
**KIT**	D816, D820, N822, Y823, Exons 11, 13
**KRAS**	G12, G13, Q61, K117, A146
**MAP2K1**	Q56, K57, K59, D67, P387
**MAP2K2**	F57, Q60, K61, L119
**MET**	Y1253, Exons 13, 18
**NRAS**	G12, G13, Q61, K117, A146
**PDGFRA**	D842
**PIK3CA**	E542, E545, Q546, D549, M1043, N1044, A1046, H1047, G1049
**PTEN**	R130, R173, R233
**RET**	C634, M918
**STK11**	Q37, P281, F354

(b)

CBioPortal Gene Query list: AKT1 ALK AR BRAF CDKN2A CTNNB1 EGFR ERBB2 ESR1 EZH2 FGFR1 FGFR2 GNA11 GNAQ HRAS IDH1 IDH2 JAK1 KIT KRAS MAP2K1 MAP2K2 MET NRAS PDGFRA PIK3CA PTEN RET STK11

Supplementary Material, Table S2: Summary of detected genetic alterations

Gene / Study ID	*EGFR*	*GNA11*	*HRAS*	*IDH2*	*KRAS*	*MAP2K2*	*PIK3CA*	*RET*	*STK11*
**Non-Synonymous mutations**									
ON11									c.1062C>G (F354L)
ON12									c.1062C>G (F354L)
ON83									c.1062C>G (p.F354L)
ON80						c.178C>T (p.Q60*)			c.1062C>G (p.F354L)
ON19							c.1633G>A (E545K)		c.1062C>G (p.F354L)
ON16							c.1633G>A (p.E545K)		
ON47							c.1633G>A (p.E545K)		
ON77		c.625C>T (p.Q209*)					c.1633G>A (p.E545K)		
ON98			c.34G>A (p.G12S)				c.1633G>A (p.E545K)		
ON15					c.183A>T (Q61H)				
ON25					c.34G>A (G12S)				
ON87					c.35G>T (p.G12V)				
ON89					c.183A>C (p.Q61H)				
**Synonymous mutations**									
ON63	c.2289C>G (p.A763A) c.2508C>T (p.R836R)								
ON07				c.420G>A (p.R140R)					
ON54				c.420G>A (p.R140R)					
ON81								c.1902C>T (p.C634C)	
